# Research on Enhancing Fire Detection Performance in Ancient Architecture Under Occlusion Scenarios Based on YOLO-AR

**DOI:** 10.3390/s26041357

**Published:** 2026-02-20

**Authors:** Chen Li, Minghan Wang, Lei Lei, Honghui Liu, Kaiyin Gao, Zuoyi Wang

**Affiliations:** School of Fire Protection Engineering, China People’s Police University, Langfang 065000, China; 2024904007@cppu.edu.cn (C.L.); 2024903039@cppu.edu.cn (M.W.); 2025904005@cppu.edu.cn (H.L.); 2025909013@cppu.edu.cn (K.G.); 2025909005@cppu.edu.cn (Z.W.)

**Keywords:** ancient architecture fire detection, self-built dataset, attention mechanism, loss function, occlusion interference

## Abstract

Fire detection in ancient architecture presents significant challenges due to complex scenes and unique structural characteristics. Traditional detection methods often demonstrate limitations when addressing the specific structural idiosyncrasies of individual ancient buildings and the overlapping occlusion prevalent in architectural complexes. This paper proposes YOLO-AR, a novel fire detection algorithm based on an improved YOLOv8 framework. By embedding the Convolutional Block Attention Module (CBAM) at the end of the backbone network, the algorithm enhances its capability to capture key features of flames and smoke. Furthermore, the Repulsion Loss function is introduced to explicitly optimize bounding box localization accuracy in occluded and dense scenarios. Experiments conducted on a self-constructed ancient architecture dataset comprising 15,847 images demonstrate that YOLO-AR outperforms mainstream comparative algorithms in terms of Precision, Recall, and mean Average Precision (mAP). Specifically, the detection precision reached 90.7%, and the recall rate improved to 89.7%. This study provides an efficient and reliable visual detection solution for early warning systems in ancient architecture, offering significant value for cultural heritage preservation.

## 1. Introduction

China possesses a rich history where countless traditional ancient buildings have been preserved as shared cultural treasures of humanity. However, these architectural ensembles face unique fire safety challenges. Structurally, they are predominantly supported by wooden columns, with complex roofs constructed from flammable components such as beams, rafters, purlins, brackets, and roof boards—materials typically rated at low fire resistance levels (Level 3 or 4). Furthermore, the interiors are often adorned with combustible materials, including hanging silk fabrics, prayer flags, canopy umbrellas, and embroidered textiles, which significantly increase the fire load. Under traditional human monitoring, the time gap between a fire outbreak and its detection often wastes the critical time window for intervention, leading to the irreversible destruction of heritage dating back centuries. Although traditional heat and smoke detectors are widely used, they are prone to high rates of false negatives and false positives within the high-ceilinged, complex frameworks of ancient buildings. Moreover, these conventional sensors fail to meet the urgent demands for the rapid, precise response required for cultural heritage preservation. Consequently, integrating artificial intelligence-based visual detection has become a crucial method for safeguarding these structures.

With the advancement of video processing, early fire warning based on image analysis has emerged as a research hotspot. Early studies, such as those by Huang et al. [1], integrated rough sets (RS) and support vector machines (SVM) to improve prediction accuracy, while Chino et al. [2] proposed static image detection using color and texture classification in superpixel regions. Recently, deep learning and Convolutional Neural Networks (CNNs) have revolutionized this field. For instance, Luo et al. [3] proposed a CNN-based algorithm utilizing smoke motion characteristics to address the limitations of static feature fusion. Since Redmon et al. [4] introduced the YOLO (You Only Look Once) algorithm, it has become the mainstream approach due to its speed. Subsequent iterations have consistently improved performance; notably, YOLOv3 utilized the Darknet-53 backbone [5] and feature pyramid networks to effectively handle complex backgrounds, as demonstrated by Park et al. [6] in the context of nighttime fire detection. YOLOv5, characterized by its lightweight architecture, offers rapid inference and effective multi-scale prediction. However, it exhibits limitations in detecting distant or irregular targets—specifically dynamic fire sources—due to its reliance on predefined anchors and susceptibility to localization instability.

YOLOv8, introduced by Ultralytics in 2023 as a state-of-the-art object detection model, represents a comprehensive evolution of YOLOv5. Adopting an Anchor-Free paradigm and a Decoupled Head structure, it eliminates predefined anchors to enhance generalization. Structurally, it introduces ELAN-inspired C2f modules to optimize gradient flow and integrates Distribution Focal Loss (DFL) with Varifocal Loss (VFL), significantly refining localization precision for hard samples while maintaining computational efficiency. Recent works, such as Huang et al. [7], have also explored embedding the Convolutional Block Attention Module (CBAM) into YOLOv5 to refine feature extraction. Beyond general-purpose improvements, recent research has increasingly focused on solving detection challenges in specific, complex real-world scenarios through the construction of specialized datasets. For instance, Zunair et al. [8] proposed RSUD20K, a large-scale dataset for road scene understanding that specifically addresses the difficulties of object detection in densely crowded and occluded environments. Their work validates that standard models often struggle to generalize to scenes with extreme clutter and varying environmental conditions without targeted data support. However, despite these advancements, existing algorithms still exhibit limitations in ancient architectural scenarios. Field investigations reveal that conventional models struggle with the unique “occlusion interference” caused by overlapping beams, brackets, and dense building clusters, often leading to missed detections or inaccurate localization in complex scenes.

To address these challenges, this paper proposes YOLO-AR, an innovative fire detection algorithm tailored for ancient structures. Our method integrates the CBAM attention mechanism at the terminal layer of the backbone network to enhance the capture of key flame and smoke features against complex backgrounds. Crucially, we introduce the Repulsion Loss function to explicitly optimize bounding box regression. This loss function minimizes the distance to the target while maximizing the repulsion between predicted boxes and surrounding non-target boxes, thereby solving the issue of detection errors in occluded and densely populated scenes. The model’s performance is validated on a specialized, self-built dataset of 15,847 images covering diverse ancient architectural scenarios. The main contributions of this research are as follows:

1. Designed and validated an improved fire detection framework, YOLO-AR, specifically addressing the distinct characteristics of ancient architecture (e.g., structural occlusion and texture interference).
2. Validated the design rationality of introducing the CBAM attention mechanism at the end of the backbone network based on feature level analysis, effectively enhancing key feature representation in complex backgrounds.
3. Adapted and applied the Repulsion Loss function to specifically resolve the bounding box regression challenges under the occlusion of dense ancient architectural components.
4. Dataset Construction: A specialized dataset tailored for ancient building fire scenarios was constructed to conduct systematic experimental validation.

## 2. Materials and Methods

This section surveys the theoretical foundations and technological landscape relevant to the study. It critically analyzes the YOLOv8 network architecture, the principles of the Convolutional Block Attention Module (CBAM), and the mechanism of the Repulsion Loss function, thereby establishing the methodological groundwork for the algorithmic enhancements proposed in subsequent sections.

### 2.1. Visual Fire and Smoke Detection Algorithms

The evolution of fire detection technology has transitioned from traditional sensing mechanisms to advanced computer vision methodologies. Early visual approaches primarily relied on hand-crafted feature extraction. For instance, Chino et al. utilized color and texture features to classify flames in static images, while Huang integrated rough set theory with Support Vector Machines (SVM) to enhance prediction accuracy. However, these traditional methods often demonstrated limited generalization capabilities in complex scenarios. With the advent of deep learning, Convolutional Neural Networks (CNNs) have emerged as the dominant paradigm. Luo et al. leveraged CNNs to extract smoke motion features, significantly mitigating false positive rates. Subsequently, object detection algorithms represented by the YOLO series gained widespread adoption due to their superior end-to-end real-time performance. Specifically, Park optimized YOLOv3 for nocturnal fire detection, and Huang incorporated the Convolutional Block Attention Module (CBAM) into YOLOv5 to refine flame detection precision. Despite these advancements, existing algorithms continue to struggle with inaccurate localization and missed detections, particularly in scenarios typical of ancient architecture characterized by complex structural occlusions and high-density interference.

To further mitigate interference in complex environments, researchers have introduced various advanced network architectures. EFFNet [9], for example, significantly improved feature representation for fire detection through an efficient feature fusion network. While SmokeAgent [10] proposed an agent-based reinforcement learning framework to optimize detection strategies. In contrast to these approaches, the YOLO-AR proposed in this study is specifically tailored to address the unique structural occlusion challenges inherent in ancient architectural environments.

### 2.2. YOLOv8 Network Architecture

The overall architecture of YOLOv8 adheres to the advanced Backbone–Neck–Head design paradigm. Input images first undergo multi-level feature extraction via the Backbone network (CSPDarknet), generating feature maps at various scales. These feature maps are subsequently fed into the Neck component, which utilizes a PAN-FPN structure for feature fusion. This process effectively integrates deep semantic information with shallow positional details. Finally, the Head component adopts a mainstream decoupled design, incorporating an anchor-free mechanism to independently execute classification and regression tasks, thereby significantly enhancing detection performance [11].

**Figure 1:**
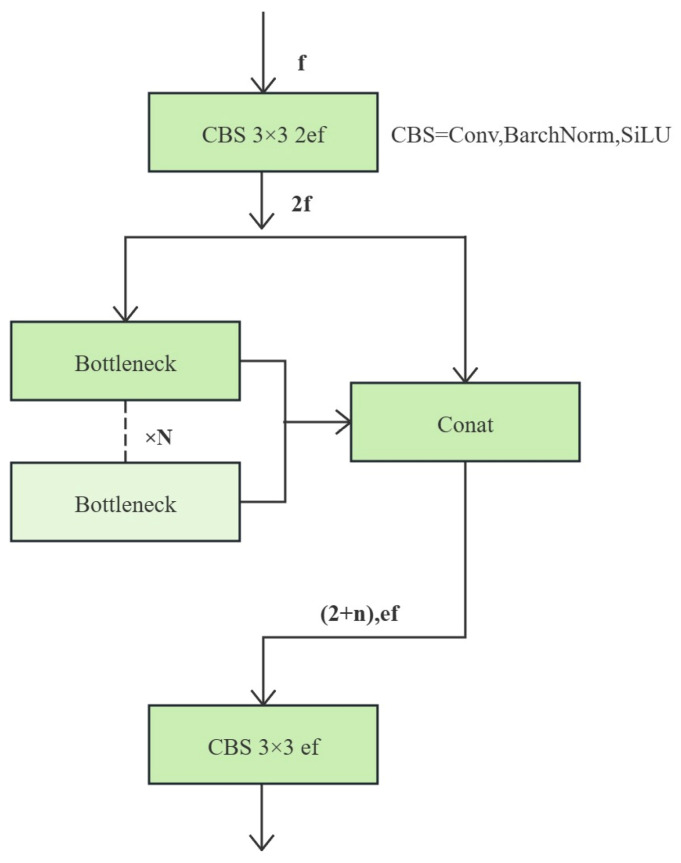
Structure of the C2f Module.

(1) Backbone Improvements: The C2f (Cross Stage Partial Fully Connected) module within the backbone is a lightweight component designed as an enhancement to the C3 (Cross Stage Partial Block 3) module used in YOLOv5 [12]. The C2f module aligns features from different intervals, achieving high model inference speed and recognition accuracy without significantly increasing computational complexity. Its structure is illustrated in Figure 1.

Additionally, YOLOv8 retains the Spatial Pyramid Pooling-Fast (SPPF) module from YOLOv5. By cascading multiple max-pooling layers, this module converts feature maps of arbitrary sizes into fixed-length feature vectors, thereby improving algorithmic accuracy.

(2) The Neck Component: The Neck component integrates multi-scale features extracted by the Backbone. YOLOv8 employs a Path Aggregation Network-Feature Pyramid Network (PAN-FPN) architecture to facilitate both top-down and bottom-up feature fusion. This bidirectional fusion mechanism ensures that the feature maps output by the Neck possess precise localization information.

(3) The Head Component: The Head component is the critical module responsible for the network’s final predictions. YOLOv8 replaces the anchor-based coupled head architecture of YOLOv5 with a decoupled head design. This approach has been proven to effectively mitigate conflicts between classification and regression tasks, allowing the model to focus on each specific learning process. This separation significantly enhances detection accuracy, particularly for classification. Furthermore, the adoption of an anchor-free detection mechanism simplifies the training process by eliminating complex anchor box clustering and matching computations, reducing the number of hyperparameters and enhancing the model’s versatility.

### 2.3. Attention Mechanism

**Figure 2:**
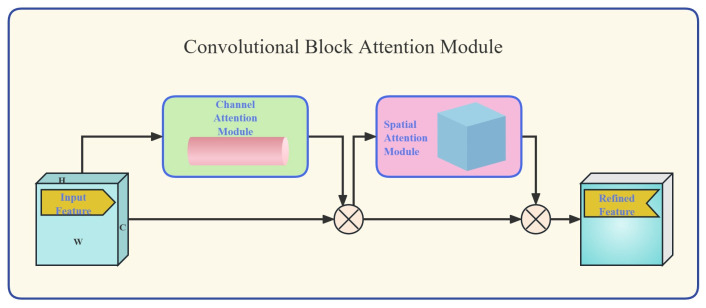
Structure of the CBAM.

The Convolutional Block Attention Module (CBAM) [13] is a lightweight attention module. Its core principle involves selectively focusing the network algorithm on information-rich regions within feature maps through attention mechanisms, thereby enhancing feature discriminability. CBAM comprises two independent submodules: the Channel Attention Module (CAM) and the Spatial Attention Module (SAM). Its complete structure is illustrated in Figure 2.

The CAM is designed to capture key features of the target object. In the specific context of fire detection in ancient buildings, the CAM enhances the color characteristics of the fire, effectively distinguishing flames from the complex architectural background. When spectral features corresponding to specific flame wavelengths are detected, the relevant channels are activated, while irrelevant background colors are suppressed. The calculation formula for CAM is presented in Equation (1) [13].

(1) $$\begin{array}{c} M_{c}=\sigma \left(MLP\left(AvgPool\left(F\right)\right)\right)+MLP\left(MaxPool\left(F\right)\right)\\ =\sigma \left(W_{1}\left(W_{0}\left(F_{avg}^{c}\right)\right)\right)+W_{1}\left(W_{0}\left(F_{max}^{c}\right)\right) \end{array}$$

In this equation, *σ* denotes the Sigmoid activation function, while **W_0_** and **W_1_** represent the weights of the two layers within the Multi-Layer Perceptron (MLP). The simultaneous deployment of Average Pooling (AvgPool) and Max Pooling (MaxPool) is designed to leverage their complementary strengths. Specifically, MaxPool is adept at extracting salient features such as textures and boundaries—for instance, the sharp tips of flames and the stratified edges of smoke. Conversely, AvgPool is essential for retaining background context and preserving global information. This synergy facilitates the precise capture of subtle fire signatures, even against the complex structural backdrops typical of ancient architecture.

Subsequently, the Spatial Attention Module (SAM) generates a spatial attention map based on the output from the Channel Attention Module (CAM), as defined in Equation (2) [13]:

(2) $$\begin{array}{c} M_{s}\left(F\right)=\sigma \left(f^{7\times 7}\left(\left[AvgPool\left(F\right);MaxPool\left(F\right)\right]\right)\right)\\ \;\;\;\;\;=\sigma \left(f^{7\times 7}\left(\left[F_{avg}^{S};F_{max}^{S}\right]\right)\right) \end{array}$$

In this equation, *f*^7×7^ denotes a convolution operation with a kernel size of 7 × 7.

### 2.4. Repulsion Loss Function

**Figure 3:**
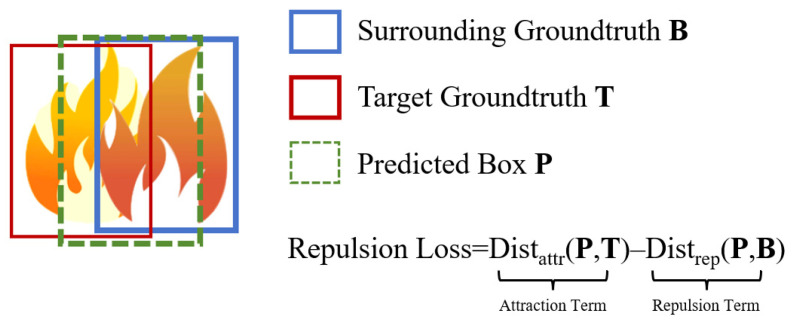
Schematic of Repulsion Loss functionality.

The intricate arrangement of Dougong (bracket sets) and flying eaves in ancient architectural complexes frequently creates severe occlusion, while the superposition of multiple fire sources often leads to bounding box localization drift. Under such conditions, reliance solely on the standard Intersection over Union (IoU) loss proves inadequate for effectively distinguishing dense targets. To mitigate this, this study introduces Repulsion Loss [14] as a substitute for the traditional regression loss. Repulsion Loss aims to maximize the proximity of the predicted bounding box **P** to the true target box **T** while simultaneously maximizing the distance between **P** and other true boxes **B** surrounding **T**. Its principle is illustrated in Figure 3.

The total loss function, *L_Rep_*, is defined as shown in Equation (3):

(3) $$L=L_{Attr}+\alpha *L_{RepGT}+\beta *L_{RepBox}$$

In this equation, *L_Attr_* represents the attraction loss term. *L_RepGT_* and *L_RepBox_* denote the repulsion losses with respect to ground-truth boxes and predicted boxes, respectively. These terms constrain the predicted bounding box to maintain distance from surrounding non-target ground-truth objects and other predicted boxes. The hyperparameters *α* and *β* balance the contribution of the repulsion terms relative to the attraction term. To determine their optimal values, we conducted a sensitivity analysis using a grid search strategy on the validation set, evaluating combinations of *α*, *β* in ∈ {0.1, 0.5, 1.0}. The experimental results indicated that the model is sensitive to these weights. Specifically, setting *α* or *β* to values ≥ 1.0 resulted in training instability and slower convergence, as the large penalty for bounding box proximity overwhelmed the attraction loss responsible for localization. Conversely, lower values failed to provide sufficient repulsion force to separate overlapping predictions in dense ancient architectural scenes. Consequently, this study adopts *α* = 0.5 and *β* = 0.5, which offered the best trade-off between training stability and occlusion handling capability, achieving the highest mAP.

(1) Attraction Term

The primary objective of the Attraction Term is to constrain the predicted bounding box to align as closely as possible with its designated ground-truth target. The formulation is given by:

(4) $$L_{Attr}=\frac{\sum_{P\in P_{+}}Smooth_{L1}\left(B^{P},G_{Attr}^{P}\right)}{\left|P_{+}\right|}$$

In this equation, $P_{+}$ denotes the set of all positive sample proposals assigned to ground-truth targets, and $\left|P_{+}\right|$ represents the cardinality of this set. For each proposal **P** in $P_{+}$, the regressed predicted bounding box is denoted as **B^P^**. **G^P^***_Attr_* refers to the designated ground-truth target for proposal **P**, specifically the ground-truth box exhibiting the maximum Intersection over Union (IoU) with **P**. The Smooth_L1_ function is employed to robustly compute the regression error between the two bounding boxes. By minimizing this term, the model ensures that the predicted boxes tightly envelop the actual flame regions within the ancient architectural context.

(2) Repulsion Term

The Repulsion Term comprises two distinct components: Repulsion against Ground-Truth (RepGT) and Repulsion against Predicted Boxes (RepBox). The RepGT Loss is designed to prevent the predicted box from shifting towards surrounding non-target ground-truth objects. It is calculated as follows:

(5) $$L_{RepGT}=\frac{\sum_{P\in P_{+}}Smooth_{ln}\left(IoG\left(B^{p},G_{Rep}^{P}\right)\right)}{\left|P_{+}\right|}$$

where **G^P^***_Rep_* represents the adjacent non-target ground-truth box that the predicted box **B^P^** must repel; specifically, it is the ground-truth box with the highest overlap with **P**, excluding the designated target. *IoG*(**B**, **G**) is defined as the Intersection over Ground-truth.

The *Smooth_ln_* function adopted in this formulation is a smooth logarithmic function that is continuously differentiable within the interval (0, 1). It is designed to amplify the penalty for overlapping regions and is defined as follows:

(6) $$Smooth_{ln}=\left\{\begin{array}{cc} -\ln\left(1-x\right) & x\leq \delta \\ \frac{x-\delta }{1-\delta }-\ln\left(1-x\right) & x>\delta \end{array}\right.$$

In this formulation, *δ* ∈ [0, 1) represents a smoothing parameter (set to 0.5 in this study) used to modulate sensitivity to outliers. By maximizing the distance between the predicted bounding box and surrounding interfering ground-truth boxes, this term effectively mitigates false positives induced by the complex structural layouts of ancient architecture.

The RepBox Loss is designed to alleviate the sensitivity of detection results to Non-Maximum Suppression (NMS). It functions by enforcing a repulsion constraint that pushes predicted boxes associated with distinct targets apart. The calculation is formulated as follows:

(7) $$L_{RepBox}=\frac{\sum_{i\neq j}Smooth_{ln}\left(IoU\left(B^{P_{i}},B^{P_{j}}\right)\right)}{\sum_{i\neq j}1\left[IoU\left(B^{P_{i}},B^{P_{j}}\right)>0\right]+\epsilon }$$

In this formulation, **P**_i_ and **P**_j_ denote predicted bounding boxes classified as distinct targets. The term *IoU* (P_i_, P_j_) quantifies the Intersection over Union between the two predictions. The notation 1[·] represents the indicator function, taking the value of 1 when the condition is satisfied and 0 otherwise. Additionally, *ϵ* is a small constant included to ensure numerical stability by preventing division by zero. The incorporation of this loss term enables YOLO-AR to effectively disentangle overlapping flame instances, thereby reducing the rate of missed detections.

## 3. Proposed Method

The architecture and implementation of the YOLO-AR algorithm, specifically tailored for ancient structures, are detailed herein. The discussion clarifies the rationale behind embedding the CBAM attention mechanism at the backbone’s terminal layer and explains how the Repulsion Loss function is incorporated to optimize bounding box regression in scenarios characterized by severe occlusion and dense targets.

### 3.1. Enhanced YOLOv8-CBAM Framework

Current academic perspectives vary regarding the optimal layer for embedding the CBAM within neural network architectures. Specifically, the placement of the attention module is often contingent upon the specific functional objectives of the application. Some approaches [15] advocate for embedding CBAM after the C2f module at every level—a pervasive integration strategy—aiming to maximize recognition accuracy irrespective of computational cost.

However, an analysis of the YOLOv8 network architecture reveals three distinct feature levels, each serving a specific purpose:

1. Early Layers: These layers capture general, low-level visual features such as edges, corners, color patches, and texture gradients [16]. These features are ubiquitous across all visual tasks.
2. Middle Layers: At this stage, the network begins combining low-level features into more complex components, distinguishing distinct texture patterns and simple geometric structure [17].
3. Deep Layers: In these layers, features become highly abstracted and systematized [18]. The network no longer focuses on isolated edges or color patches but instead captures systematic, macro-level features synthesized from the preceding layers.

In the specific context of fire detection, the shallow (early) stage presents significant ambiguity; the edges of flames are often indistinguishable from the edges of objects reflecting sunlight, and the gradients of smoke are highly similar to those of clouds and aerosols. Similarly, at the intermediate level, flames and background regions with similar hues or edge shapes remain difficult to differentiate. Consequently, prematurely embedding CBAM into low-level feature layers may cause the attention mechanism to be misled by a vast number of irrelevant details, introducing significant noise rather than useful signals [19]. This theoretical hypothesis is partially validated by the ablation experiments presented in Section A.

Furthermore, the core function of the Spatial Pyramid Pooling-Fast (SPPF) module is to capture multi-scale contextual information through pooling kernels of varying size [20]. This is crucial for addressing the extreme scale variations inherent in flames and smoke. If unweighted features are directly fed into the SPPF, the module will indiscriminately pool and fuse all features, including those containing strong background interference.

**Figure 4:**
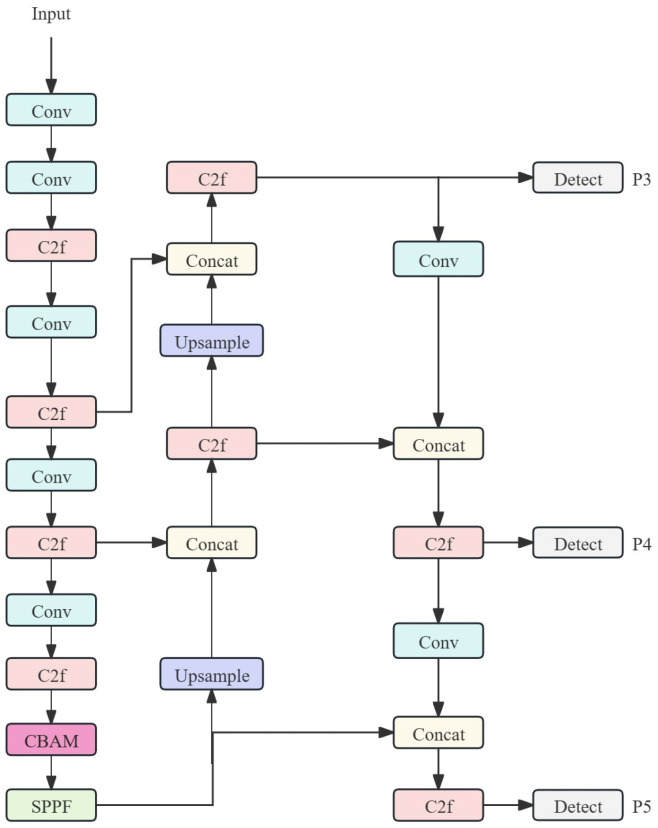
Diagram of the CBAM Embedding Position.

As previously established, feature maps at the end of the backbone contain highly refined and rich high-level semantic information [21]. At this endpoint, feature map dimensions have been significantly reduced through multiple downsampling stages. Although the channel count remains 1024, the computational overhead of CBAM’s fully connected layers and 7 × 7 convolutions at this reduced resolution offers the optimal balance between computational cost and performance enhancement. This approach leverages minimal computational resources to achieve maximum performance gains. Compared to a full-network embedding strategy, this single-point embedding significantly controls model capacity growth, preserving the model’s lightweight nature and generalization capabilities. This makes the proposed framework particularly suitable for fire detection scenarios where data availability may be limited. The structure of this embedding position is illustrated in Figure 4.

### 3.2. Introduction of Repulsion Loss

Obstruction interference in ancient building fires exhibits pronounced scene specificity, primarily manifesting in three typical scenarios:

1. Structural Obstruction: Architectural components such as beams, brackets, and eaves physically block the fire source, resulting in incomplete flame and smoke features;
2. Environmental Occlusion: Static or dynamic objects, such as artificial hills or dense tree foliage, obstruct the monitoring field of view;
3. Feature Occlusion: This arises from smoke diffusion creating layered obstructions or blending with the background textures and colors of ancient architecture.

These forms of occlusion often intertwine, making fire feature extraction and recognition significantly more challenging than in standard scenarios.

**Figure 5:**
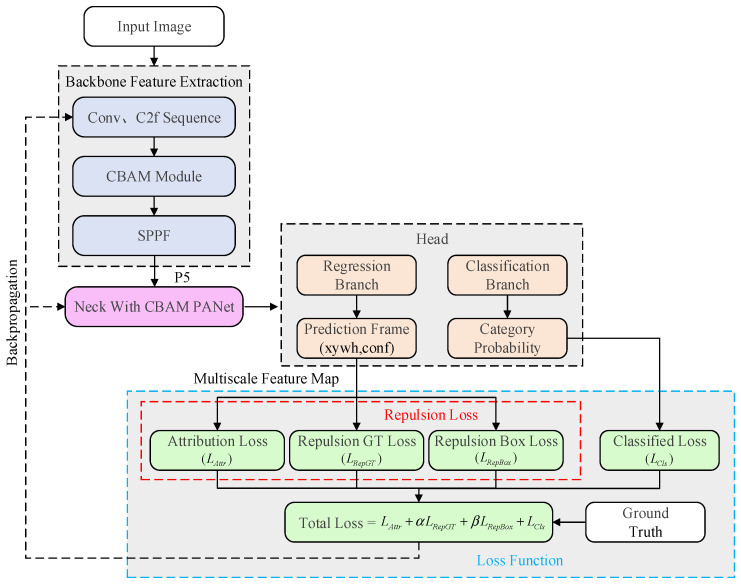
Core Flowchart of the Loss Function Calculation.

To achieve precise detection of dense and occluded fires, this paper introduces a key enhancement to the YOLOv8 loss function by supplementing the original CIoU Loss with Repulsion Loss. We integrated the Repulsion Loss function into the existing loss framework to form a synergistic system for feature enhancement and loss optimization alongside the YOLOv8-CBAM architecture. While retaining the original CIoU Loss and Distribution Focal Loss (DFL), this architecture incorporates the RepGT and RepBox components of Repulsion Loss. These components address, respectively, the repulsion relationship between predicted boxes and neighboring non-target ground truth boxes, and the mutual repulsion between overlapping predicted boxes. Serving as the optimization core during training, the Repulsion Loss module receives predictions from the detection head, calculates the total loss by integrating ground truth labels, and guides parameter updates through backpropagation. This enables precise localization and separation of bounding boxes in scenarios characterized by occlusion and dense targets. The workflow is illustrated in Figure 5.

Addressing Dense and Obscured Targets Traditional loss functions, such as standard IoU Loss [22], tend to generate large prediction boxes that cover multiple distinct fire points when handling densely overlapping flames, leading to missed detections (false negatives). Simultaneously, flames are often partially or completely obscured by smoke, forming blurry targets with weak features. In ancient architectural settings, precise localization of ignition points is critical. The Repulsion Loss term introduced in this study constrains the model to generate independent and disjoint prediction boxes for each fire point.

Concurrently, the CBAM (specifically the SAM component) focuses on potential glows or high-temperature areas within smoke, enhancing these faint yet critical features. The repulsive effect of the Repulsion Loss effectively suppresses multiple blurred, overlapping false predictions caused by smoke obstruction, compelling the model to output a single, high-confidence prediction result. When combined with CBAM’s enhancement of local flame features, the improved model achieves effective segmentation and counting of distinct ignition points, significantly boosting recall and localization accuracy in dense fire scenarios. This approach guarantees high recall rates while substantially reducing false alarms.

To integrate Repulsion Loss into the YOLOv8 framework, we extended the *v8DetectionLoss* class within the ultralytics library’s *loss.py* module. Specifically, an additional computational branch was introduced following the calculation of standard bounding box regression losses (IoU Loss and DFL). The input parameters for this module include the predicted box coordinates **P**, ground truth box coordinates **G**, and the foreground mask *fg_mask*.

During each training iteration, the repulsion loss is computed as follows: First, the IoU matrix between all predicted and ground truth boxes within the current *batch* is calculated via the *pairwise_bbox_iou* function. Second, the *L_RepGT_* and *L_RepBox_* are computed using the *Smooth_Ln_* function, with the smoothing parameter *σ* set to 0.5. Finally, these terms are weighted and summed according to Equation (3). The weight hyperparameters are fixed at *α* = 0.5 and *β* = 0.5.

## 4. Experiments and Results

To evaluate the proposed model’s efficacy, this section presents a comprehensive validation using a specialized dataset. It details the construction of the self-built ancient architecture fire dataset, specifies the experimental configuration and evaluation metrics, and offers a systematic analysis of comparative and ablation studies to verify the model’s performance advantages.

### 4.1. Experimental Setup

#### 4.1.1. Dataset Construction

**Figure 6:**
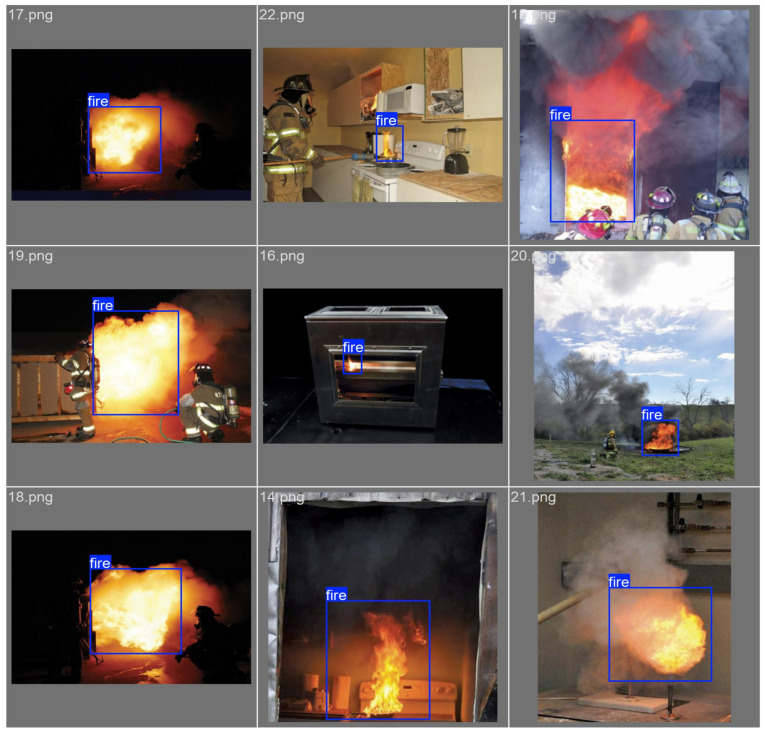
Image detection results extracted from public datasets.

To accurately evaluate the model’s performance in complex ancient architecture scenes, this study constructed a dataset specifically for ancient architecture fire detection. The dataset consists of two parts:

Multiple publicly available flame and smoke detection datasets were collected, such as FASDD [23] and the D-Fire dataset [24]. From these sources, fire images with backgrounds including architecture, forests, and laboratories were selected to enhance the model’s generalization capability and improve the robustness of fire image detection accuracy across different application scenarios, as shown in Figure 6.

To address the scarcity of ancient architectural scenes in public datasets, a proprietary dataset was constructed by collecting images from multiple historic sites, including the Forbidden City, the Summer Palace, and Youshun Temple. This dataset encompasses diverse lighting conditions, smoke densities, and occlusion scenarios. Open flames were ignited at Youshun Temple during field experiments, conducted with management approval and under stringent safety protocols. Safety and Ethical Protocols: To ensure the absolute preservation of cultural heritage, all data acquisition involving open flames was conducted under rigorous safety protocols. The experiments were performed in designated open areas exterior to the primary timber structures of Youshun Temple. Written authorization was obtained from both the local Cultural Heritage Bureau and the Fire Department prior to experimentation. Throughout the process, professional firefighters and fire-suppression equipment were stationed on-site. It is important to note that the fire sources were ignited on isolated simulation tracks and were strictly separated from the actual ancient architectural fabric.

**Figure 7:**
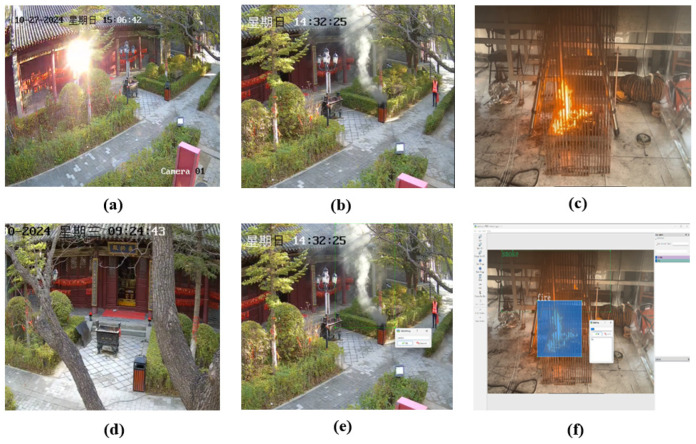
(**a**,**d**) Image acquisition experiments for negative samples under reflective interference and interference from objects resembling fire sources, respectively. (**b**,**e**) Smoke image acquisition and annotation under specific wind speed interference conditions. (**c**,**f**) Fire source image acquisition from laboratory experiments simulating grid fence obstructions.

For positive sample collection, fires were ignited at various locations on the structures using a range of materials. Lighting conditions varied between strong light, weak light, and neutral light. Smoke images were captured at different concentrations under both windy and calm conditions. To generate negative samples, simulated reflective illumination was employed, and models approximating the colors and shapes of flames were strategically placed to test false positive rates. As shown in Figure 7. It is crucial to emphasize that to circumvent the issue of “Ambiguous Supervision” arising from unlabeled positive samples, all negative samples underwent a rigorous dual-verification process by human annotators. This ensured that the images contained solely interfering background elements, with an absolute absence of actual flame or smoke instances. Within the YOLO training framework, these unannotated images were treated as pure background samples. Consequently, any candidate proposals generated by the model on such images were strictly categorized as False Positives and penalized via the Objectness Loss. Furthermore, drawing upon recent advancements in domain-specific object detection—such as the strategies employed by Succulent-YOLO [25] and UDD-YOLO [26] for handling small targets and distractors in complex backgrounds—we validated that the incorporation of pure background negative samples is indispensable for enhancing the model’s robustness in uncontrolled environments.

This study defines the detection task as a two-class object detection problem. Specifically, the detection targets are explicitly categorized into two independent classes: “Fire” and “Smoke”. During the annotation process, for images containing both fire and smoke, the two types of targets were labeled independently, allowing for bounding box overlap. When capturing images under flame-obscuring interference conditions, the research team employed two methods: obstruction by continuous pedestrian movement and obstruction by fixed architectural structures.

**Figure 8:**
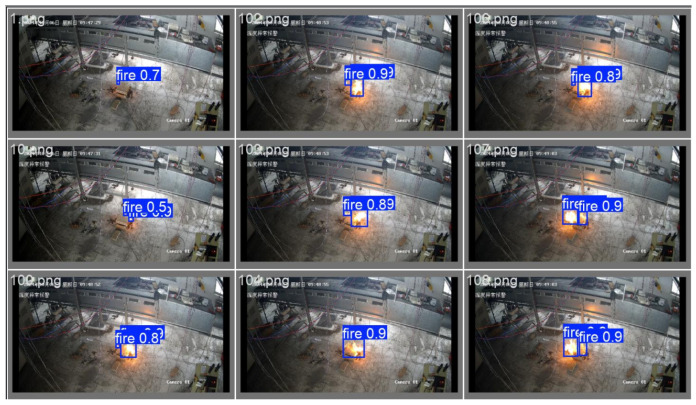
Images of specially processed combustion materials extracted from video footage and incorporated into the dataset for model training and testing.

To enhance the dataset’s suitability for ancient architectural scenarios, the research team pre-treated wood blocks to simulate the combustion properties of aged timber. Natural resins and partial extracts were removed using solvents such as acetone [27]. These blocks subsequently underwent thermal treatment at 80–160 °C for durations ranging from tens to hundreds of hours, simulating the effects of centuries of natural thermal aging. The treated blocks were then assembled using traditional mortise-and-tenon joinery techniques. Artifacts such as books, albums, silk textiles, and knitted fabrics also underwent corresponding technical treatments to impart similar simulated antique combustion properties. Following these preparations, the materials were ignited experimentally to extract their stage-by-stage combustion characteristics. Continuous video recording captured these stages, with selected frames extracted to create a historical building fire dataset. This dataset better reflects the combustion imagery specific to materials and structural conditions found in ancient architectural settings, as shown in Figure 8.

Ultimately, the dataset comprises 15,847 annotated images, consisting of 9512 flame samples and 6335 smoke samples. To ensure the fairness of the evaluation and prevent data distribution bias, we employed a strict Stratified Sampling strategy when dividing the Training, Validation, and Test sets. This strategy ensures that the ratio of self-built ancient architecture data to public data remains consistent across all subsets (approximately [e.g., 7:3]). Furthermore, strict de-duplication was performed to ensure that no highly similar sequence frames exist between the training and test sets, effectively avoiding potential data leakage risks. Positive samples were meticulously annotated using the LabelImg tool to mark individual, non-contiguous detection targets. Conversely, negative interference samples remained unlabeled; including these background images without annotations is a strategic method to enhance the model’s robustness against interference and reduce false positive rate [28]. The detailed partitioning of the dataset is presented in Table 1.

Prior to training, all images underwent uniform preprocessing operations, including resizing to 640 × 640 pixels. To simulate complex real-world visual variations and enhance model robustness, data augmentation techniques were applied, including Mosaic augmentation [29], random rotation, brightness, contrast, and saturation adjustments, as well as random HSV color space dithering [30].

#### 4.1.2. Experimental Environment and Implementation Details

All experiments were conducted under identical software and hardware conditions to ensure result comparability.

Hardware Environment: All experiments were conducted on a high-performance mobile workstation powered by an Intel Core Ultra 7 155H CPU and an NVIDIA GeForce RTX 4060 Laptop GPU (8GB VRAM). To eliminate the impact of stochasticity on the ablation results and ensure that comparisons between configurations are both equitable and statistically significant, a strictly standardized training protocol was implemented across all experiments. Specifically, all models were initialized with identical weights, utilized consistent hyperparameter settings, and operated under a fixed random seed (Random Seed = 0) to guarantee deterministic training behavior. Consequently, the performance disparities observed—particularly those related to the positioning of the CBAM—are primarily attributable to architectural modifications rather than random fluctuations in initialization.

Software Environment: The experiments utilized the Windows 11 operating system, the PyTorch 2.7.0 deep learning framework accelerated with CUDA 12.6, and the Python 3.9 programming language.

Model training employed the following key parameters: an input resolution of 640 × 640, a batch size of 16, 4 data loading threads, an initial learning rate of 0.01, and a total of 200 training epochs. For the Repulsion Loss, a grid search determined that optimal model performance was achieved with an attraction weight of *α* = 0.5 and a repulsion weight of *β* = 0.5.

To ensure a rigorous and fair assessment of practical inference capabilities, all latency and Frame Per Second (FPS) benchmarks were conducted under identical hardware configurations. The testing protocol utilized a Batch Size of 1 and a consistent input resolution of 640 × 640. Furthermore, half-precision (FP16) inference was enabled to simulate real-world deployment scenarios on edge devices. The testing procedure followed a strict benchmarking protocol. Initially, 50 warm-up iterations were executed to ensure the GPU reached a stable operating state. Subsequently, continuous inference was performed on 1000 images from the test set, and the average latency per frame was calculated. The reported Frames Per Second (FPS) values are based on end-to-end inference time, which explicitly encompasses the entire pipeline: image pre-processing, model inference, and Non-Maximum Suppression (NMS) post-processing. This metric was selected to authentically reflect the model’s response speed in practical application scenarios. The model’s computational complexity, specifically the parameter count and Floating Point Operations (FLOPs), was quantified using the thop library for PyTorch.

### 4.2. Evaluation Metrics

To comprehensively evaluate model performance, metrics widely recognized in the object detection field are generally adopted [31], specifically Precision (*P*), Recall (*R*), *F*1 Score, Average Precision (*AP*), and Mean Average Precision (mAP). *P* measures the proportion of samples correctly predicted as positive cases by the model, reflecting the accuracy of the detection. *R* measures the proportion of all true positive cases correctly predicted, used to assess the comprehensiveness of the model’s detection. The *F*1 Score is a harmonic mean evaluation metric combining *P* and *R*. *AP* calculates the area under the precision-recall curve for a specific category. Mean Average Precision (mAP) averages the *AP* values across all categories, serving as a core metric for evaluating the model’s overall detection accuracy, which is jointly determined by *P* and *R*.

The formulas for Precision and Recall are as follows:

(8) $$Precision=\frac{TP}{TP+FP}$$

(9) $$Recall=\frac{TP}{TP+FN}$$

The Average Precision formula is as follows:

(10) $$AP=\int_{1}^{0}Precision\left(Recall\right)d\left(Recall\right)$$

The *F*1 score, a harmonic mean evaluation metric for precision *P* and recall *R*, is calculated as follows:

(11) $$F1=\frac{2\times Precision\times Recall}{Precision+Recall}$$

The formula for calculating the Mean Average Precision value (mAP) is as follows:

(12) $$M_{AP}=\frac{1}{C}\sum_{i=1}^{C}A_{Pi}$$

During each training iteration, samples are categorized into positive and negative samples. If a positive sample is correctly identified as positive, it is termed a True Positive (TP). If a positive sample is incorrectly identified as negative, it is termed a False Negative (FN). If a negative sample is incorrectly identified as positive, it is termed a False Positive (FP). If a negative sample is correctly identified as negative, it is termed a True Negative (TN). The confusion matrix is shown in Table 2.

### 4.3. Experimental Results and Analysis

To comprehensively evaluate the performance of the proposed YOLO-AR model in ancient building fire detection tasks, this section presents a systematic analysis from both quantitative and qualitative dimensions. First, comparative experiments with traditional object detection algorithms on the same test dataset validate the comprehensive advantages of the proposed method in terms of detection accuracy (mAP), recall. Second, the visualization of detection results demonstrates the model’s robustness under conditions of complex occlusions, color interference, and small-scale fire scenarios. Finally, ablation studies clarify the individual contributions and synergistic benefits of the CBAM attention mechanism and the Repulsion Loss function.

#### 4.3.1. Detection Performance Comparison

**Figure 9:**
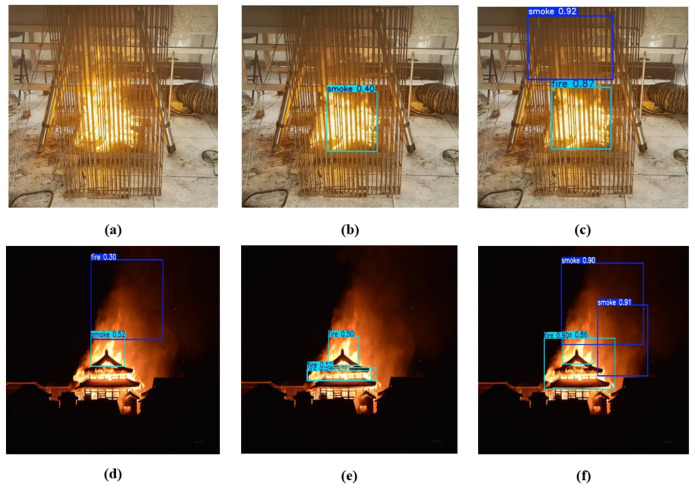
Visualization of qualitative analysis results on the independent test set. This figure demonstrates the recognition performance under partially occluded fire sources and ancient architecture night scenes. (**a**,**d**) Detection results of the Faster R-CNN model (showing missed detections); (**b**,**e**) Detection results after only replacing with Repulsion Loss; (**c**,**f**) Inference results of the YOLO-AR model. As shown, YOLO-AR generates more accurate bounding boxes and higher confidence scores in complex occlusion and low-light environments, verifying the model’s robustness in real-world scenarios.

This section evaluates the detection performance of the YOLO-AR model in real-world scenarios by comparing its recognition of detection targets across different environments. To visually demonstrate the model’s detection capabilities under complex occlusion and interference conditions, this study selected representative partially occluded fire sources from ancient building fire scenarios and conducted a visual comparison analysis with nighttime ancient building fire scenes. The results are presented in Figure 9. In partially occluded scenarios (Figure 9a–c), flames are partially obscured by architectural elements such as window frames and columns. The traditional Faster R-CNN model [32] (Figure 9a) failed to fully detect the obscured flame area, resulting in missed detections. The model incorporating only Repulsion Loss (Figure 9b) partially mitigated missed detections of overlapping targets but still exhibited inaccurate bounding box localization and low confidence scores. In contrast, the proposed YOLO-AR model (Figure 9c) accurately identifies visible portions of obscured flames and generates high-confidence bounding boxes, demonstrating robust occlusion resistance. In nighttime fire scenes (Figure 9d–f), where dim lighting and interference from firelight and artificial light coexist, Faster R-CNN (Figure 9d) exhibits false positives and localization errors. The model enhanced solely with Repulsion Loss (Figure 9e) improves false detection suppression but still suffers from insufficient recall for distant, small-sized flames. Finally, the YOLO-AR model (Figure 9f), leveraging CBAM to enhance flame luminance features and Repulsion Loss to precisely constrain bounding boxes, achieves stable flame detection in low-light environments. These visualizations further validate YOLO-AR’s robust performance and practicality in complex scenarios such as occlusion conditions.

#### 4.3.2. Comparative Analysis Experiments

To empirically validate the efficacy and performance superiority of the proposed method, a comprehensive comparative analysis was conducted on the YOLO-AR model. Comparisons were performed against the Faster R-CNN model, the baseline YOLOv8n model, and the YOLOv8 model embedded with the CBAM attention mechanism, utilizing the identical dataset. The experimental data are summarized in Table 3.

As detailed in Table 3 (Performance Comparison of Different Detection Models), YOLO-AR achieved an overall mean Average Precision (mAP) of 91.6%. It is crucial to emphasize that this study prioritizes the model’s Holistic Perception of fire incidents within ancient architectural contexts, rather than focusing on the isolated segmentation performance of individual categories. This methodological choice is necessitated by the unique nature of these environments (e.g., temples), where benign smoke from ceremonial incense burning is prevalent. In such scenarios, smoke detection in isolation—devoid of spatial association with a fire source—lacks practical utility for early warning systems and is prone to triggering false positives. Consequently, the comprehensive mAP is adopted as the definitive metric for evaluating the model’s reliability in distinguishing actual threats from complex environmental interferences.

The experimental data indicates that YOLO-AR has a parameter count of only 3.02 M and a computational cost of 8.20 G FLOPs. Compared to the two-stage detection algorithm Faster R-CNN (41.35 M Params, 180.0 G FLOPs), YOLO-AR compresses the parameter size to 7.3% and reduces the computational cost to 4.5% of Faster R-CNN, while achieving a significant 20.5% improvement in mAP@0.5.

The proposed YOLO-AR model—YOLOv8 integrated with both CBAM and Repulsion Loss—achieved the best performance among all models. Compared to the CBAM-only model, YOLO-AR maintained high accuracy (90.7%) while significantly boosting recall from 86.5% to 89.7%. Despite the use of a heterogeneous dataset during the training phase, the model demonstrated robust generalization capabilities, achieving a high mean Average Precision (mAP) of 91.6% on a test set predominantly composed of ancient architectural scenarios (approximately 70%). This key metric demonstrates that incorporating Repulsion Loss effectively addresses missed detections caused by flame occlusion or dense flame clusters. By enabling the model to capture more true positive samples, it substantially enhances detection comprehensiveness.

Ultimately, the YOLO-AR model achieved a Mean Average Precision (mAP) of 91.6% and an F1 score of 90.0%, both representing the highest values across all tested models. This fully demonstrates the effectiveness of combining the CBAM attention mechanism with the Repulsion Loss function in the field of ancient building fire detection. CBAM focuses on critical information at the feature level, enhancing recognition quality in single detections, while Repulsion Loss optimizes bounding box layout through geometric constraints, improving accuracy in capturing partially occluded targets within complex scenes [33]. Working synergistically, they jointly drive the model’s core evaluation metrics—mAP and F1 score—to new heights. In terms of detection accuracy and recall, the proposed method surpasses the comparison models, validating its advanced and robust nature.

#### 4.3.3. Ablation Studies

**(A) Impact of CBAM Embedding Positions**

To investigate the impact of different embedding positions of the CBAM attention module within the YOLOv8 network on ancient building fire detection performance, this study designed ablation experiments for four embedding strategies [34]: shallow layer (CBAM-S), middle layer (CBAM-M), deep layer (CBAM-D), and the end of the backbone network (CBAM-B). The experimental results are summarized in Table 4. As observed in the table, different CBAM embedding positions exert varying effects on model performance.

CBAM-S (Shallow Embedding) places the CBAM at the shallow feature extraction stage of the network. Since shallow features primarily contain low-level visual information such as edges and color patches, prematurely introducing attention mechanisms can easily cause the model to be distracted by numerous irrelevant details—such as sunlight reflections and complex building textures—leading to noise amplification and the dilution of key fire features. Therefore, while CBAM-S achieves a slight improvement in precision (89.2%), its mAP (87.5%) and recall (84.8%) are marginally lower than the baseline model. This indicates that shallow embedding may introduce redundant noise in complex scenes, hindering the model’s overall ability to capture fire targets.

CBAM-M (Mid-level Embedding) integrates CBAM at the mid-level feature stage. By this point, the network has preliminarily fused low-level features to form more complex texture and shape representations. The attention mechanism begins to focus on flame color regions and smoke diffusion patterns, resulting in slight improvements over the baseline in mAP (88.2%), precision (89.8%), and recall (85.5%). This indicates that mid-level embedding has a positive effect, though the enhancement is limited, suggesting this feature level still contains substantial intermediate structural information unrelated to fires.

CBAM-D (Deep Embedding) embeds the module at the deep feature layer, where features are highly abstracted and rich in semantic information [35]. Attention mechanisms enable more effective differentiation between flames, smoke, and complex backgrounds at this high-level semantic stage. Experimental results show that CBAM-D achieves significant improvements in precision (90.2%), recall (86.0%), and mAP (88.8%), demonstrating that applying attention at the deep level substantially enhances the model’s ability to distinguish key fire features.

CBAM-B (Embedding at Backbone Endpoint) places CBAM at the end of the backbone network, immediately before features enter the Neck for multi-scale fusion. Features at this position retain refined high-level semantic information while maintaining moderate spatial resolution [36]. At this stage, the attention mechanism maximally focuses on the most discriminative regions—such as the luminous core of flames and the diffusion center of smoke—while effectively suppressing background interference. Experimental results demonstrate that CBAM-B achieves optimal performance in precision (89.8%), recall (86.5%), and mAP (89.2%), significantly performing better than other embedding strategies. This validates that applying attention to high-level semantic features more precisely guides the network to focus on regions most relevant to fire detection, thereby achieving higher detection accuracy and recall in complex occlusion and interference scenarios.

In summary, the embedding position of CBAM must align with feature abstraction levels [37]. Embedding CBAM at the end of the backbone network maximizes the advantages of the attention mechanism in feature enhancement and interference suppression while minimizing computational overhead, establishing it as the optimized strategy adopted in this study.

**(B) Impact of Enhancement Modules**

To thoroughly analyze the specific contributions of the proposed enhancement modules—namely the CBAM attention mechanism and Repulsion Loss—to model performance, systematic ablation experiments were designed. Experiments were conducted under identical training settings across four distinct model configurations:

1. Baseline Model (Model 1): The original YOLOv8n architecture utilizing the standard CIoU loss function;
2. CBAM-only (Model 2): Embeds the CBAM at the end of the YOLOv8n backbone while retaining CIoU as the loss function;
3. Repulsion Loss-only (Model 3): Introduces only Repulsion Loss, retaining the original network architecture but replacing the regression loss component with Repulsion Loss;
4. Complete Model YOLO-AR (Model 4): Simultaneously embeds the CBAM and utilizes Repulsion Loss.

The experimental results are presented in Table 5.

As shown in the table, the results indicate the following:

- Using CBAM alone (Model 2) yields a significant improvement in Precision, rising from 88.6% to 89.8%. This demonstrates that CBAM effectively focuses on key features of flames and smoke while suppressing background interference, thereby enhancing single-object recognition precision. However, its mAP and Recall did not show substantial increases, indicating that feature enhancement alone remains insufficient to address false negatives in dense or occluded scenarios.
- Using Repulsion Loss alone (Model 3) yields modest gains in both Precision and Recall. This suggests Repulsion Loss optimizes bounding box distribution through geometric constraints, mitigating missed detections of overlapping objects. However, its effectiveness remains constrained by the limitations of the original feature extraction capabilities.
- The complete YOLO-AR model (Model 4) achieves optimal performance across all metrics, with particularly significant improvements in Recall and mAP. This demonstrates a clear synergistic enhancement effect between CBAM and Repulsion Loss: CBAM strengthens object discriminability at the feature level, while Repulsion Loss optimizes bounding box localization and separation capabilities at the regression level. Their combination effectively resolves detection challenges in complex scenarios such as flame occlusion and dense object distribution within ancient architectural settings.

## 5. Discussion

Experimental results indicate that CBAM significantly improves model precision, validating its anti-interference effectiveness. Concurrently, Repulsion Loss explicitly constrains prediction boxes to approach true targets while avoiding overlap with other true and predicted boxes through attraction and repulsion terms. This mechanism effectively enhances detection precision in complex, specific historical building fire scenarios. Compared with existing fire detection methods, the proposed YOLO-AR model demonstrates unique advantages in the following aspects:

1. Interference Resistance: YOLO-AR effectively suppresses structural occlusions and background color interference commonly encountered in ancient buildings;
2. Detection Comprehensiveness: The model achieves high detection comprehensiveness, significantly reducing the false negative rate of missed detections caused by dense flames or smoke occlusions;
3. Speed-Accuracy Balance: Based on the lightweight design of YOLOv8, the model maintains a high frame rate (FPS) while delivering detection accuracy superior to two-stage methods like Faster R-CNN, making it highly suitable for real-time monitoring scenarios.

Limitations and Error Analysis: Although YOLO-AR performs exceptionally well in most ancient architecture scenarios, detection errors still occur in extreme cases. The primary limitations are twofold:

1. False Positives: Common objects like red lanterns, painted walls, and strong reflections share similar color distributions with fire, occasionally leading to misclassification. Additionally, certain clouds with regular patterns may be confused with early-stage smoke.
2. False Negatives: When fire sources are at extremely long distances or are severely occluded by complex structural elements, the model may fail to capture sufficient feature information, resulting in missed detections. To address these issues, relying solely on static RGB images may have reached a bottleneck, and future work will need to incorporate temporal analysis or multimodal data.

Feasibility Analysis and Practical Deployment Recommendations: To rigorously evaluate the applicability of the proposed system for early fire warning in ancient architectural contexts, a structured analysis of deployment constraints was conducted based on the experimental findings. The key recommendations are as follows:

1. Camera Deployment Strategy: Given the unique structural complexities of ancient architecture—characterized by the occlusion from Dougong (bracket sets) and expansive overhanging eaves—a single-view perspective is insufficient for comprehensive coverage. Therefore, a dual-tier monitoring strategy is recommended. This involves integrating high-vantage surveillance (to cover the panoramic view of roofs and courtyards) with low-level blind spot monitoring (installed beneath corridors to specifically target the obscured corners of bracket sets and beams). This hierarchical arrangement ensures the elimination of visual dead zones critical to fire safety.
2. Real-Time Performance and Computational Resource Allocation: Experimental benchmarks demonstrate that the model achieves an inference speed of 112 FPS on an NVIDIA RTX 4060 GPU. Considering that standard security surveillance streams typically operate at 25 FPS, a single edge server is capable of processing more than four high-definition video streams simultaneously in real-time. This efficiency fully satisfies the operational requirements for “early detection and second-level response,” making it highly viable for large-scale deployment.
3. All-Weather Environmental Adaptability: Addressing common environmental interferences such as morning mist or smoke from burning incense, the proposed model has demonstrated robust noise resistance through the incorporation of negative sample training. However, in extreme weather conditions (e.g., torrential rain or dense fog) where the effective range of visible-light cameras is compromised, it is recommended to integrate the system with Distributed Temperature Sensing (DTS) optical fibers or infrared thermal imaging devices. Such fusion creates a comprehensive, multi-modal sensing framework capable of maintaining reliability under all environmental conditions.

## 6. Conclusions

Traditional fire detection methods primarily target general indoor environments with low ceilings. In contrast, this paper specifically focuses on ancient architectural complexes, which present unique detection challenges due to their intricate structures, severe obstructions, and numerous sources of color interference. While conventional thermal and smoke detectors remain widely used as general monitoring and alarm tools, they are prone to false negatives and false positives in complex scenarios involving multiple interference sources. Moreover, they fail to meet the increasingly urgent demands for precise identification and rapid response required for the protection of cultural heritage. To address the challenges specific to ancient architecture—including structural obstructions, complex backgrounds, and difficulties in small object recognition—this paper developed a custom dataset tailored for ancient building fire detection. It proposes YOLO-AR, a detection algorithm based on an enhanced YOLOv8 framework. The key contributions and conclusions are as follows:

1. Strategy Optimization and Validation: Instead of pursuing purely algorithmic novelty, this paper focuses on the effective integration of existing advanced components within a specific domain. Through validation, embedding a CBAM attention module at the end of the YOLOv8 backbone enhances feature extraction capabilities for flames and smoke through dual-level attention mechanisms (channel and spatial), effectively suppressing complex background and lighting interference. Furthermore, replacing the original regression loss with Repulsion Loss introduces a repulsion term to optimize bounding box localization accuracy in dense, occlusion-prone scenarios, significantly reducing false negatives for overlapping targets.
2. Experimental Validation: Systematic validation was conducted on a self-built ancient building fire dataset comprising 15,847 images. The results demonstrate that the YOLO-AR model outperforms Faster R-CNN, the original YOLOv8n, and a baseline model equipped with CBAM alone across key metrics, including Precision, Recall, mAP, and F1 score. Notably, significant improvements are observed in Recall (89.7%) and mAP (91.6%), indicating that the model achieves comprehensive target capture capabilities while maintaining high precision.
3. Ablation Analysis: Ablation experiments further validate the synergistic gains derived from combining CBAM and Repulsion Loss. CBAM primarily enhances single-object recognition quality, while Repulsion Loss optimizes multi-object bounding box separation capabilities. Their integration demonstrates exceptional performance in occluded and dense flame scenarios.
4. Practical Significance: This study provides an efficient and reliable visual detection solution for the early warning of fires in ancient buildings, achieving a favorable balance between detection speed, accuracy, and interference resistance. It actively promotes the preventive conservation of humanity’s ancient cultural heritage.

Moving forward, our research group will continue exploring multimodal data fusion, model lightweighting, and cross-scenario transfer learning to further enhance the algorithm’s applicability and robustness under extreme weather conditions and within resource-constrained environments.

## Figures and Tables

**Table 1:** Dataset Partitioning.

Dataset	Number of Images	Flame Samples	Smoke Samples
Training Set	11,093	6659	4434
Validation Set	2374	1425	949
Test Set	2380	1428	952
Total	15,847	9512	6335

**Table 2:** Confusion Matrix.

Labeled Type	Predicted	Confusion Matrix
Positive	True	TP
Positive	False	FN
Negative	False	FP
Negative	True	TN

**Table 3:** Performance Comparison of Different Detection Models.

Method	Params (M)	FLOPs (G)	FPS	Precision (%)	Recall (%)	mAP@0.5 (%)
Faster R-CNN	41.35	180.0	24	72.1%	69.8%	71.1%
BoWFire	-	-	-	68.5%	71.2%	65.4%
Fire-YOLOv3	61.9	154.6	58	82.3%	78.5%	80.1%
YOLOv8n	3.01	8.19	120	88.6%	85.4%	88.0%
YOLOv8 + CBAM	3.02	8.20	115	89.8%	86.5%	89.2%
YOLO-AR	3.02	8.20	112	90.7%	89.7%	91.6%

**Table 4:** Comparative Analysis of CBAM Embedding Strategies.

Model	mAP (%)	Precision (%)	Recall (%)	F1	FPS
YOLOv8n	88.0	88.6	85.4	0.869	120
CBAM-S	87.5	89.2	84.8	0.869	115
CBAM-M	88.2	89.8	85.5	0.876	118
CBAM-D	88.8	90.2	86.0	0.878	116
CBAM-B	89.2	89.8	86.5	0.881	115

**Table 5:** Ablation Study on Enhancement Modules.

Specimen	CBAM	RepLoss	mAP	Precision	Recall
1	**×**	**×**	88.0%	88.6%	85.4%
2	**√**	**×**	89.2%	89.8%	86.5%
3	**×**	**√**	88.5%	90.3%	85.6%
4	**√**	**√**	91.6%	90.7%	89.7%

## Data Availability

The data presented in this study are available from the corresponding author upon request, provided that permission is granted by the authorized personnel.
